# Correlation Between Fibroblast Growth Factor 23 and Biochemical Parameters in Hemodialysis Patients With End-Stage Renal Disease

**DOI:** 10.7759/cureus.85293

**Published:** 2025-06-03

**Authors:** Barat Yusubov, Mirkhalig Javadov, Khanbaba Huseynov, Muradali Bakhshiyev

**Affiliations:** 1 Department of Nephrology, Yeni Klinika Hospital, Baku, AZE; 2 Department of General Surgery and Transplantation, Yeni Klinika Hospital, Baku, AZE; 3 Department of Extracorporeal Detoxification and Hemodialysis, Scientific Surgical Center named after Academician M. Topchubashov, Baku, AZE; 4 Department of Internal Medicine, Azerbaijan Medical University, Baku, AZE

**Keywords:** calcium, chronic kidney disease, end-stage renal disease, fibroblast growth factor 23, homocysteine, intact parathyroid hormone, phosphorus

## Abstract

Background: Fibroblast growth factor 23 (FGF-23) is one of the biomarkers that plays a role in regulating phosphate (P) levels in hemodialysis (HD) patients with end-stage renal disease (ESRD). In addition to FGF-23, intact parathyroid hormone (iPTH) and homocysteine (Hcy) also play a role in the chronic kidney disease (CKD)-related mineral bone disease (MBD) process. In this study, we evaluated the correlation of glomerular filtration rate (GFR), serum creatinine, calcium, and phosphorus levels with FGF-23, iPTH, and Hcy. These parameter values may guide us for new prognostic and treatment definition targets in HD patients with ESRD.

Methods: We retrospectively evaluated serum FGF-23, Hcy, and iPTH concentrations in 103 HD patients with ESRD. Biochemical laboratory parameter data were analyzed to determine the correlation between GFR, blood creatinine, phosphorus, and calcium levels. The inclusion criteria for this study were patients with chronic kidney disease who were undergoing HD and did not have primary cardiovascular disorders. The exclusion criteria included patients with primary cardiovascular disease undergoing HD; patients with acute renal failure; patients with acute cardiovascular failure, and patients with other pathologies undergoing HD (e.g. autoimmune and oncological diseases, patients who had undergone surgery within the last two months). Color Doppler Echocardiography and Electrocardiogram (ECG) were performed to all HD patients for cardiac evaluation. Before HD blood samples were collected and the centrifugation method was used for serum fluid collection. We used the ELISA method to record demographic data and biochemical laboratory parameter results for all patients.

Results: In 103 cases of HD patients, 65 were male (63.1%) and 38 were female (36.9%). The mean ± SD parameter result for age was 64±13.64. The detailed baseline information for biochemical laboratory parameters, including Hg, CRP, glucose, creatinine, GFR, K, Na, Ca, P, ALT, AST, iPTH, Hcy, and FGF-23, were evaluated. FGF-23 levels were positively correlated with serum P (r=0.78, p=0.01), iPTH (r=0.61, p=0.01), and creatinine (r=0.78, p=0.01) and negatively with GFR (r=-0.64, p=0.01). FGF-23 and Hcy showed a positive linear correlation (r=0.65, p=0.01). No statistically significant correlation was found with Ca values (r=-0.12, p>0.05).

Conclusion: There is no single biomarker that can completely predict CKD progression and follow-up. Changes in FGF-23, iPTH, and Hcy levels together and correlation with other biochemical laboratory parameters may be the earliest markers for HD patients. These parameter values together can be used to guide strategies for the prognostic issues and early treatment management.

## Introduction

Chronic kidney disease (CKD)-related mineral bone abnormalities (MBD) are one of the leading causes of mortality in hemodialysis (HD) patients with end-stage renal disease (ESRD) [1]. Pathological changes in phosphocalcic metabolism plays an important role in increasing CVD risk factors among patients with CKD [2]. Phosphocalcic metabolism conditions showed us new prognostic biomarkers for earlier medical or surgical interventions. The CKD-MBD syndrome is characterized by increased or decreased values of mineral elements and bone metabolism changes caused by CKD. These changes include abnormal calcium (Ca), phosphorus (P) metabolism, and abnormal parathyroid hormone (PTH) levels [1,2].

Fibroblast growth factor 23 (FGF-23) is a major regulator of phosphate (P) metabolism. It is secreted by osteocytes and is associated with a higher risk of mortality in HD patients with ESRD [3-5]. Some studies have reported that there is no association between a single value of FGF-23 and all-cause mortality. However, increasing FGF-23 concentrations can identify patients at risk for mortality [5,6]. FGF-23 inhibits the reabsorption of P by the renal tubules. Additionally, it inhibits PTH secretion and active vitamin D3 synthesis, thereby limiting intestinal P absorption [5-8].

FGF-23 also plays an effective role in the pathological processes of CVD, including left ventricular hypertrophy, myocardial injury, coronary atherosclerosis, and vascular calcification. Recent studies have reported direct FGF-23 effects on the heart myocardium, and elevated plasma levels of FGF-23 have been associated with negative CVD outcomes such as arrhythmias and heart failure [8,9].

FGF-23 is progressively elevated in ESRD patients on HD. This mechanism is explained by the feedback response to persistent hyperphosphatemia and deficiency of the co-factor klotho. The relationship between FGF-23 levels and CVD, such as cardiorenal syndrome and MBD, in HD patients is not yet clear [10-12]. 

Renal hyperparathyroidism (rHPT) develops during the early stages of renal failure with high risks of bone fractures, CVD, and death [13,14]. PTH and FGF-23 increase as kidney function declines, and when patients reach kidney failure, they fail to exert their phosphaturic effects. This process leads to hyperphosphatemia and further elevations of both hormones. FGF-23 levels should be measured in clinical practice targeting to control it just as with PTH levels. The target range levels for intact PTH (iPTH) remain a matter of debate. Further research is needed to determine the optimal iPTH level ranges in HD patients with ESRD [13-16].

Recent articles have shown that homocysteine (Hcy) is a predictive and prognostic marker for HD patients with CKD. In patients with CKD, Hcy levels were found higher compared to normal population. Some articles have reported that hyperhomocysteinemia (Hhcy) has recently been recognized as an independent risk factor for developing CVD, with a rate of 85-90%. Notably, when combined with biochemical laboratory parameters such as creatinine, albumin, calcium, and CRP, Hhcy can be used as a prognostic marker for ESRD. As a result, it has been recommended to monitor Hcy levels in all CKD HD patients [17-20].

Research has shown that no single biomarker alone can predict CKD progression and follow-up prognosis. Glomerular filtration rate (GFR) and creatinine are follow-up markers for kidney failure situation [21-25]. CVD pathological processes, like cardiorenal syndrome, are the leading cause of death in HD patients with ESRD [26-29].

There have been numerous studies and publications in which FGF-23, Hcy, and iPTH values are correlated together with other biochemical laboratory parameters in HD patients with ESRD. In this study, we evaluated the correlation between FGF-23, iPTH, and Hcy levels and other biochemical laboratory parameters in Azerbaijani patients, the majority of whom belong to the Caucasian race.

## Materials and methods

We retrospectively evaluated 103 ESRD patients from a single center who underwent dialysis for at least three months. Ethical approval was obtained from the Institutional Ethics Committee. The following inclusion criteria defined as patients with chronic kidney disease without primary cardiovascular disorders during HD. The exclusion criteria were defined as patients with primary cardiovascular disease who underwent HD, patients with acute renal failure, patients with acute cardiovascular failure, and patients with other pathologies who underwent HD (e.g. autoimmune and oncological diseases, patients who had undergone surgery within the last two months). Only a few patients were on phosphate binders, vitamin D, calcimimetics, etc., and most of the patients were at stage 5. We decided that their exclusion from the analysis had no impact on our results.

Major clinical information, including age, gender, primary disease, comorbid diseases, and data of all patients, were collected. In addition to the classic biochemical laboratory parameter analyses, such as creatinine, urea, sodium, potassium, calcium, phosphorus, AST, ALT, and CRP, new parameters such as FGF-23, iPTH, and Hcy levels were retrospectively evaluated from data that have been widely used in some centers in recent years. Additionally, GFRs (ml/min) were calculated as a routine standard procedure, as performed in previous similar research [1,2]. Color Doppler Echocardiography and Electrocardiogram (ECG) were performed on all HD patients for cardiac evaluation.

We divided all patients into two groups: the first group (main group) with high FGF-23 and Hcy levels and the second group (comparison group) with no increase in Hcy and FGF-23 levels. The planned examinations were performed for both groups. Additionally, FGF-23 values correlated with iPTH and Hcy values.

We calculated renal functions using the CKD-EPI formula by calculating estimated glomerular filtration rate (eGFR) and categorizing patients into five stages, as established by the K/DOQI guidelines, similar to previous research [1,2]. GFR stages were defined as >90, 60-89, 30-59, 15-29, and <15 ml/min/1.73 m^2^ [21].

Before HD, blood samples were collected, and the centrifugation method was used for serum fluid collection. Creatinine, Ca, P, and other parameters of standard biochemistry elements were measured using standard procedures at our laboratory. Enzyme-linked immunosorbent assay (ELISA) was used to measure FGF-23 serum levels. The human FGF-23 ELISA kits used in this study were manufactured by Immutopics International (San Clemente, CA, USA; #60-6600). For normal reference value measurements of FGF-23, 28 healthy volunteers with similar age and gender distributions, who were not receiving any medication or treatment, were selected. The mean ± SD FGF-23 value in 28 healthy controls was 17.28 ± 6.44 (pg/mL).

Electrochemiluminescence immunoassay (ECLIA, PTH Cobas®, Roche) was used to measure iPTH levels within the normal range of 15-65 pg/ml, standard procedure for determining normal control values. The Fluorescence Polarization Immunoassay (FPIA) method was used to measure total Hcy concentration, with a normal reference range of 5-15 µmol/L. Target reference range for phosphorus (1.13-1.78 mmol/l), calcium (2.1-2.4 mmol/l), and intact PTH (150-300 pg/ml) in CKD patients were evaluated (K/DOQI guidelines) [15].

In terms of data analysis, descriptive statistics were presented with mean and standard deviation values. Frequency and percentage values were provided for proportional variables. Chi-square (χ^2^) analysis was performed for proportional evaluations of the study groups. Non-parametric tests were selected because the distributions were not suitable for normal distribution, as determined by the Shapiro-Wilk test during the examination of normality tests for the measurements of the groups in the study. The Mann-Whitney U test was used to examine the difference between the control and main groups in the study. Spearman correlation was applied to examine the relationships between FGF-23 levels and other parameters. The parameters were analyzed using IBM SPSS Statistics for Windows, Version 25.0 (Released 2017; IBM Corp., Armonk, New York, United States). All the statistical tests were two-sided; the significance level was p<0.05.

## Results

We retrospectively evaluated a total of 103 HD patients. The baseline clinical and laboratory characteristics of the patients are shown in Table 1. In total of 103 HD patients, 65 were male (63.1%) and 38 were female (36.9%). The mean ± SD age of the study population was 64±13.64 years. The detailed baseline information of biochemical parameters, including Hg, CRP, glucose, creatinine, GFR, K, Na, Ca, P, ALT, AST, iPTH, and Hcy, FGF-23 were evaluated and presented in Table 1. 

**Table 1:** Baseline clinical and laboratory characteristics (n=103). Descriptive statistics: Quantitative data presented as mean ± SD, categorical data presented as number (percentage), and p-value <0.05 considered significant. HGB, hemoglobin; HT, hematocrit; CRP, C-reactive protein; eGFR, estimated glomerular filtration rate; ALT, alanine transaminase; AST, aspartate aminotransferase; FGF-23, fibroblast growth factor 23; iPTH, intact parathyroid hormone; Hcy: homocysteine.

Parameters	No. of patients = 103, n (%)
Gender, n (%)	
Male	65 (63.1)
Female	38 (36.9)
	Mean ± SD
Age (years)	64 ± 13.64
Height (cm)	170 ± 6.9
Weight (kg)	79 ±13.04
Vital parameters	
Systolic blood pressure (mm/Hg)	140 ± 17.83
Diastolic blood pressure (mm/Hg)	80 ± 6.13
Pulse (per minute)	83 ± 11.3
Laboratory parameters	
HGB (g/dl)	9.3 ± 11.9
HT (%)	30.4 ± 6.36
CRP (mg/l)	12.31 ± 18.67
eGFR (ml/min/1.73 m^2^)	11 ± 5.78
Creatinine (umol/l)	599 ± 242.2
Glucose (mmol/l)	6.2 ± 3.2
Urine protein (mg/dl)	150 ± 85.8
Potassium (mmol/l)	4.1 ± 0.57
Sodium (mEq/l)	142 ± 3.32
Calcium (mmol/l)	2.34 ± 3.17
Phosphorus (mmol/l)	1.69 ± 1.94
ALT (U/l)	22.1 ± 13.06
AST (U/l)	22.4 ± 12.56
FGF-23 (pg/ml)	876 ± 584.8
İPTH (pg/ml)	153 ± 93.7
Hcy (umol/ml)	20 ± 7.42

It was found that the distribution of males and females in the groups did not differ statistically (p=0.16) as presented in Table 2. It was determined that phosphorus levels differed according to the groups in the study (p=0.01). Phosphorus levels were higher in the study group. Calcium levels did not differ significantly in the main and control groups (p=0.29). iPTH levels differed according to the groups (p=0.01) and iPTH levels were higher in the main group. In the study, it was determined that GFR levels differed according to the groups (p=0.01). GFR levels were lower in the main group (Table 3).

**Table 2:** Examining the characteristics of gender distribution according to study groups (n=103). *p-value <0.05 considered significant. **Chi-square test was performed.

Patient characteristics (n=103)	Control (n=28), n (%)	Main (n=75), n (%)	p**
Male	15 (54)	50 (67)	0.16*
Female	13 (46)	25 (33)	

**Table 3:** Analysis of laboratory characteristics according to study groups (n=103). Categorical data presented as number (percentage). *p-value <0.05 considered significant. **Chi-square test was performed. Target reference range (t.r.r.) for phosphorus (1.13-1.78 mmol/l), target reference range for calcium (2.1-2.4 mmol/l), and target reference range for intact PTH (150-300 pg/ml) (K/DOQI guidelines).

Patient characteristics (n=103)	Control (n=28), n (%)	Main (n=75), n (%)	p**
Phosphorus < t.r.r.	23 (82)	20 (26.7)	0.01*
Phosphorus > t.r.r.	5 (18)	55 (73.3)	
Calcium < t.r.r.	19 (67.9)	59 (78.7)	0.29
Calcium > t.r.r.	9 (32.1)	16 (21.3)	
iPTH < t.r.r.	22 (78.5)	4 (5.3)	0.01*
iPTH > t.r.r.	6 (21.5)	71 (94.7)	
GFR > 15ml/min/1.73m^2^	23 (82)	4 (5.3)	0.01*
GFR < 15ml/min/1.73m^2^	5 (18)	71 (94.7)	

We conducted a chi-square (χ²) test to compare the biochemical parameter results of two groups: the first group consisted of 75 patients (main group) with high FGF-23 and Hcy levels and the second group consisted of 28 patients (comparison group) with no increase in FGF-23 and Hcy levels (Tables 4, 5). FGF-23 and iPTH values showed a positive linear correlation (r=0.61, p=0.01). FGF-23 and Hcy values showed a positive linear correlation (r=0.65, p=0.01). It was purely coincidental that the number of normal healthy volunteers in the group was 28.

**Table 4:** Analysis of the relationships between the changes in FGF-23 and other biochemical laboratory parameters in the groups (n=103). Spearman correlation test, * p-value <0.05 considered significant. Crea: creatinine; Ca: calcium; P: phosphorus; iPTH: intact parathyroid hormone; GFR: glomerular filtration rate.

Group			FGF-23	p
Control (n=28)	Crea	r	0.25^*^	0.01
	Ca	r	-0.18	>0.05
	P	r	-0.31	>0.05
	iPTH	r	-0.28	>0.05
	GFR	r	-0.11	>0.05
Main (n=75)	Crea	r	0.78^*^	0.01
	Ca	r	-0.12	>0.05
	P	r	0.78^*^	0.01
	iPTH	r	0.61^*^	0.01
	GFR	r	-0.64*	0.01

**Table 5:** Analysis of creatinine, calcium, phosphorus, iPTH, and GFR levels in groups (n=103). *p-value <0.05 considered significant. **Mann-Whitney U test. Quantitative data presented as X ± S.D.: mean ± SD; categorical data presented as number (n=103). Crea: creatinine; Ca: calcium; P: phosphorus; iPTH: intact parathyroid hormone; GFR: glomerular filtration rate.

Parameters	Patient group (n=103)		p**
	Control (n=28)	Main (n=75)	
	X ± SD	X ± SD	
Crea 1	389.73 ± 113.43	748.87 ± 201.49	0.01*
Crea 2	515.46 ± 209.16	697.6 ± 191.98	0.01*
Crea 3	484.21 ± 184.95	661.45 ± 211.91	0.01*
Crea 4	489.18 ± 204.52	646.11 ± 204.98	0.01*
Ca 1	4.35 ± 3.16	5.1 ± 3.18	0.09
Ca 2	4.03 ± 3.01	4.93 ± 3.23	0.34
Ca 3	4.79 ± 3.49	5.8 ± 3.42	0.11
Ca 4	4.18 ± 3.18	6.00 ± 3.05	0.06
P 1	2.22 ± 1.78	2.71 ± 2.04	0.01*
P 2	1.90 ± 1.46	3.04 ± 2.59	0.01*
P 3	2.08 ± 1.15	2.77 ± 2.10	0.01*
P 4	2.27 ± 1.55	2.90 ± 2.27	0.01*
GFR 1	17.21 ± 7.27	9.83 ± 3.38	0.01*
GFR 2	13.50 ± 7.24	10.60 ± 4.42	0.04*
GFR 3	14.00 ± 13.22	10.74 ± 4.52	0.03*
GFR 4	13.57 ± 13.28	10.75 ± 3.73	0.04*
iPTH1	121.54 ± 27.06	235.23 ± 90.97	0.01*
iPTH 2	124.25 ± 31.16	242.76 ± 98.41	0.01*
iPTH 3	135.46 ± 57.67	250.27 ± 105.28	0.01*
iPTH 4	133.32 ± 43.79	257.51 ± 112.29	0.01*

**Figure 1:**
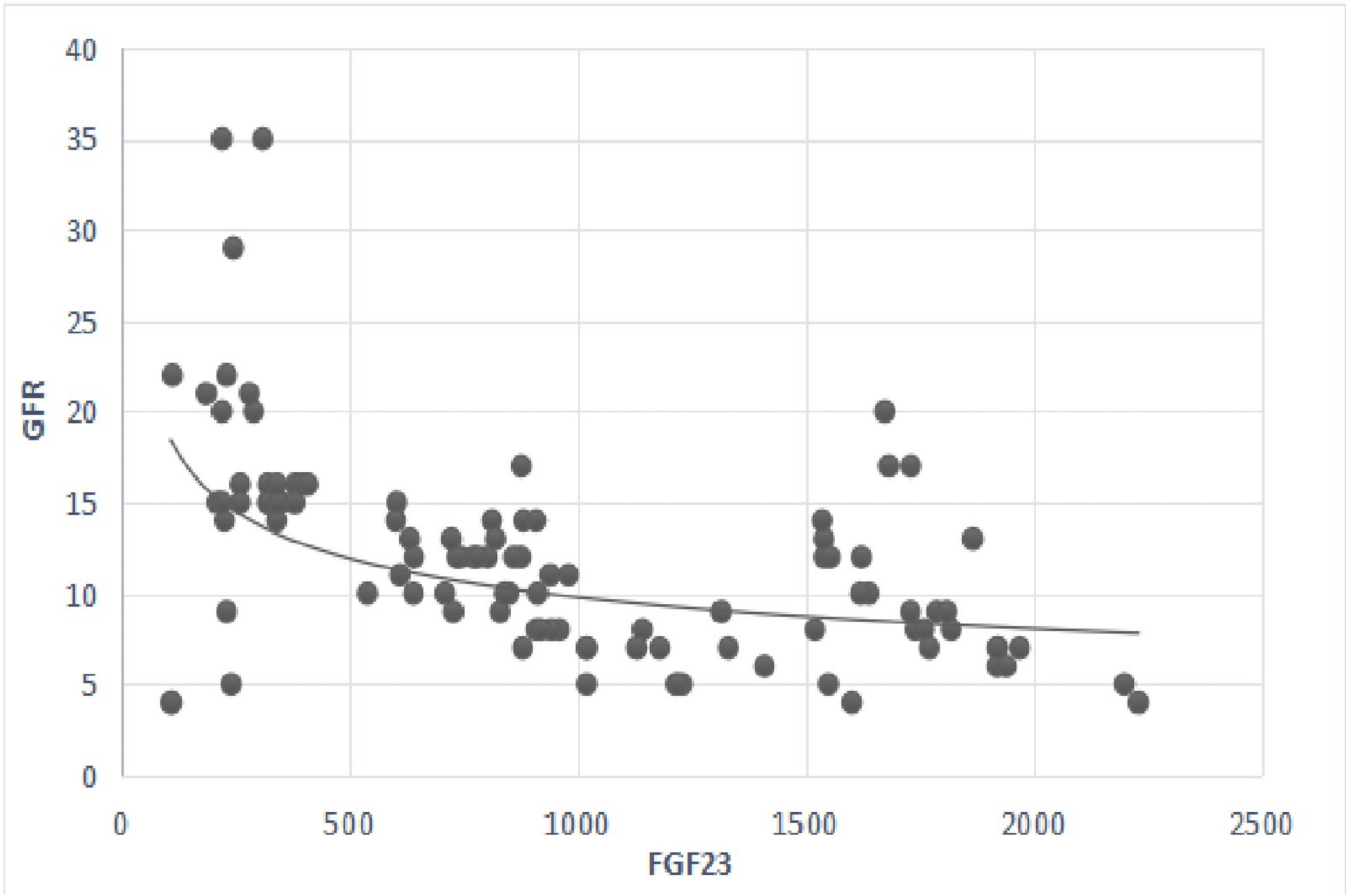
Correlation between serum levels of FGF-23 and GFR. p-value <0.05 considered significant; r=-0.64; p=0.01. GFR: glomerular filtration rate (ml/min/1.73m2); FGF-23: fibroblast growth factor 23 (pg/ml).

**Figure 2:**
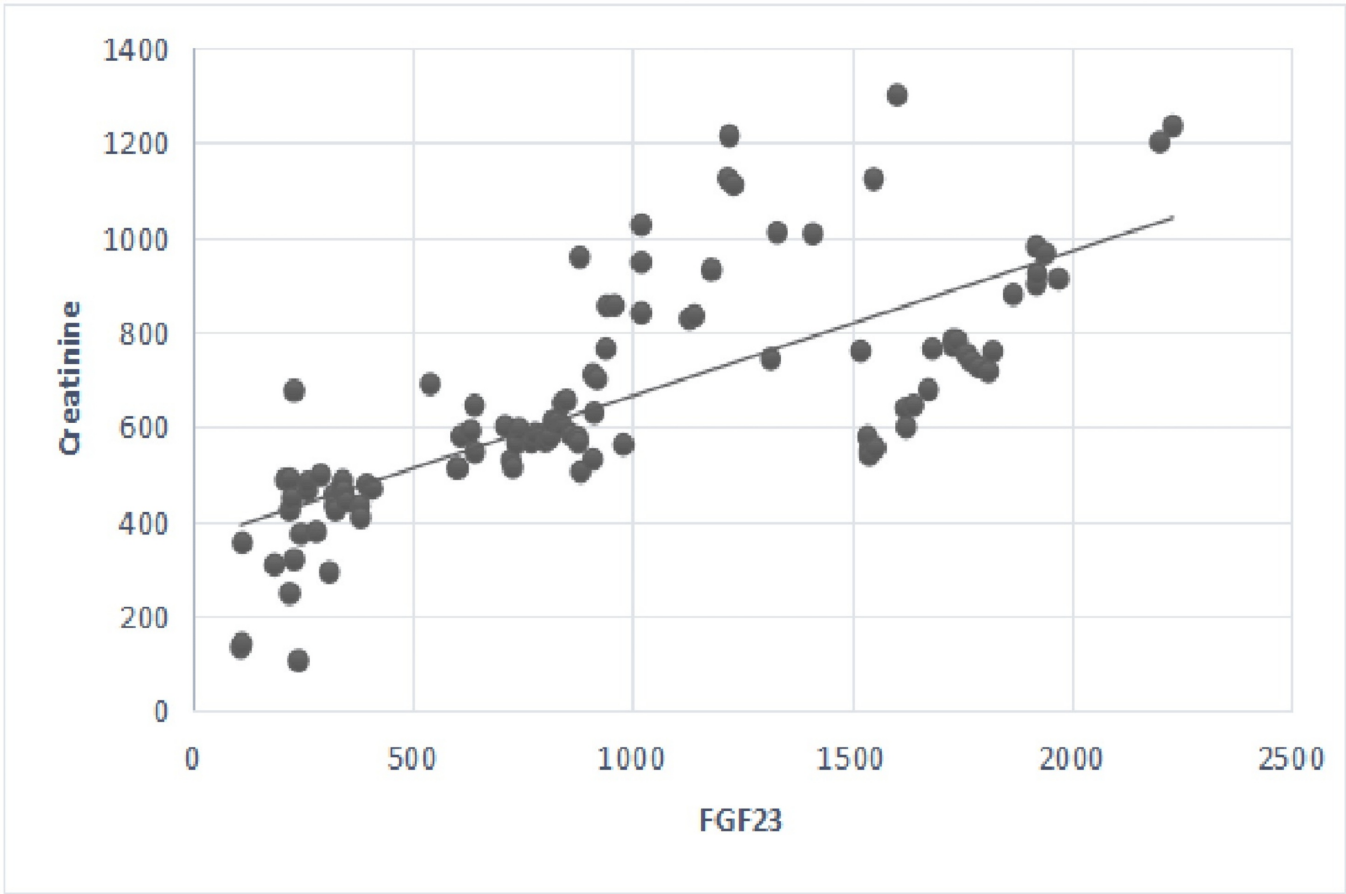
Correlation between serum levels of fibroblast growth factor 23 and serum creatinine. p-value <0.05 considered significant; r=0.78, p=0.01. Creatinine (umol/l); FGF-23: fibroblast growth factor 23 (pg/ml).

FGF-23 had a negative linear correlation with GFR values and a positive linear correlation with creatinine (Figures 1, 2).

**Figure 3:**
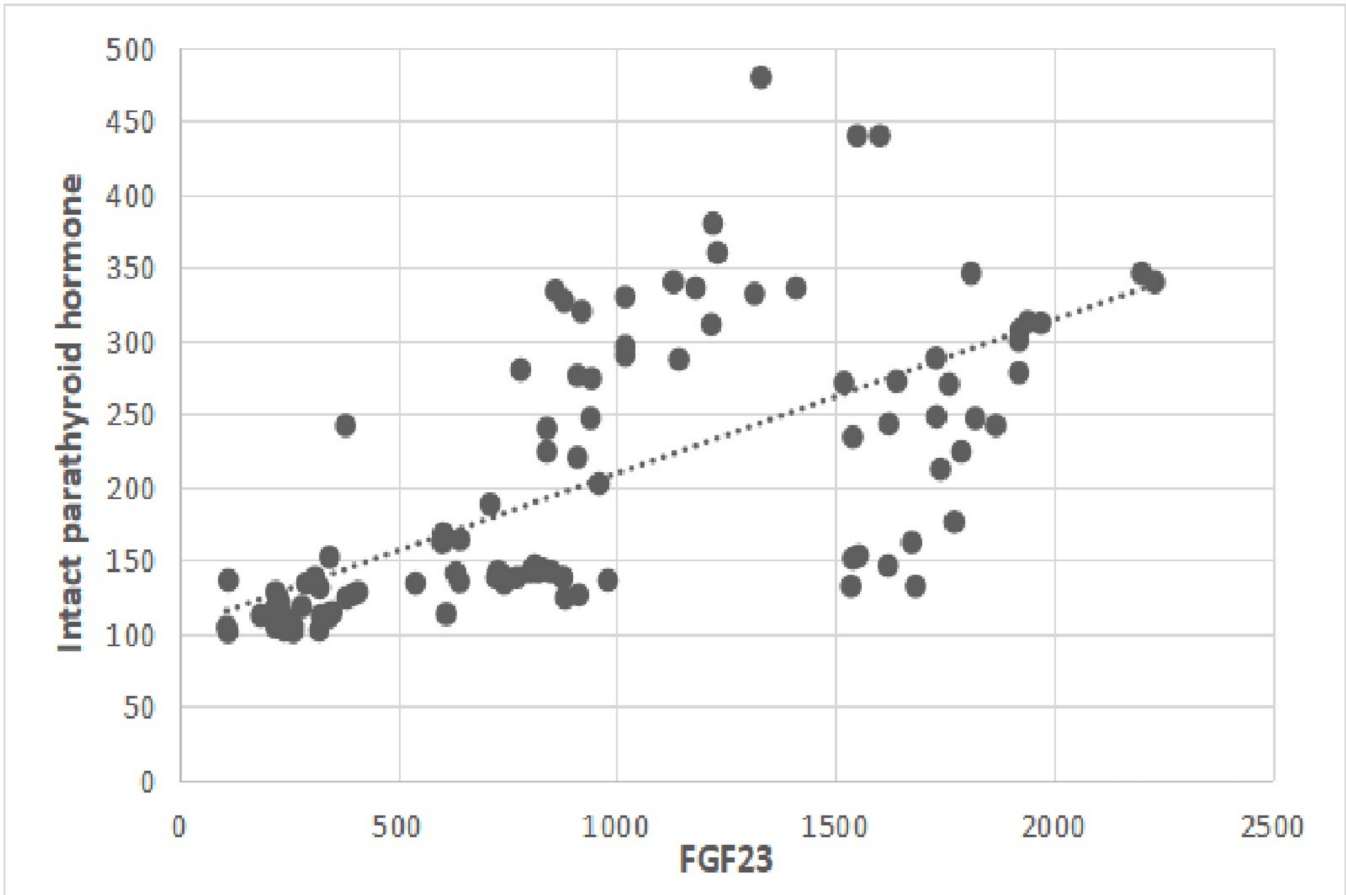
Correlation between serum levels of FGF-23 and serum levels of iPTH. p-value <0.05 considered significant; r=0.61, p=0.01. iPTH: intact parathyroid hormone (pg/ml); FGF-23: fibroblast growth factor 23 (pg/ml).

**Figure 4:**
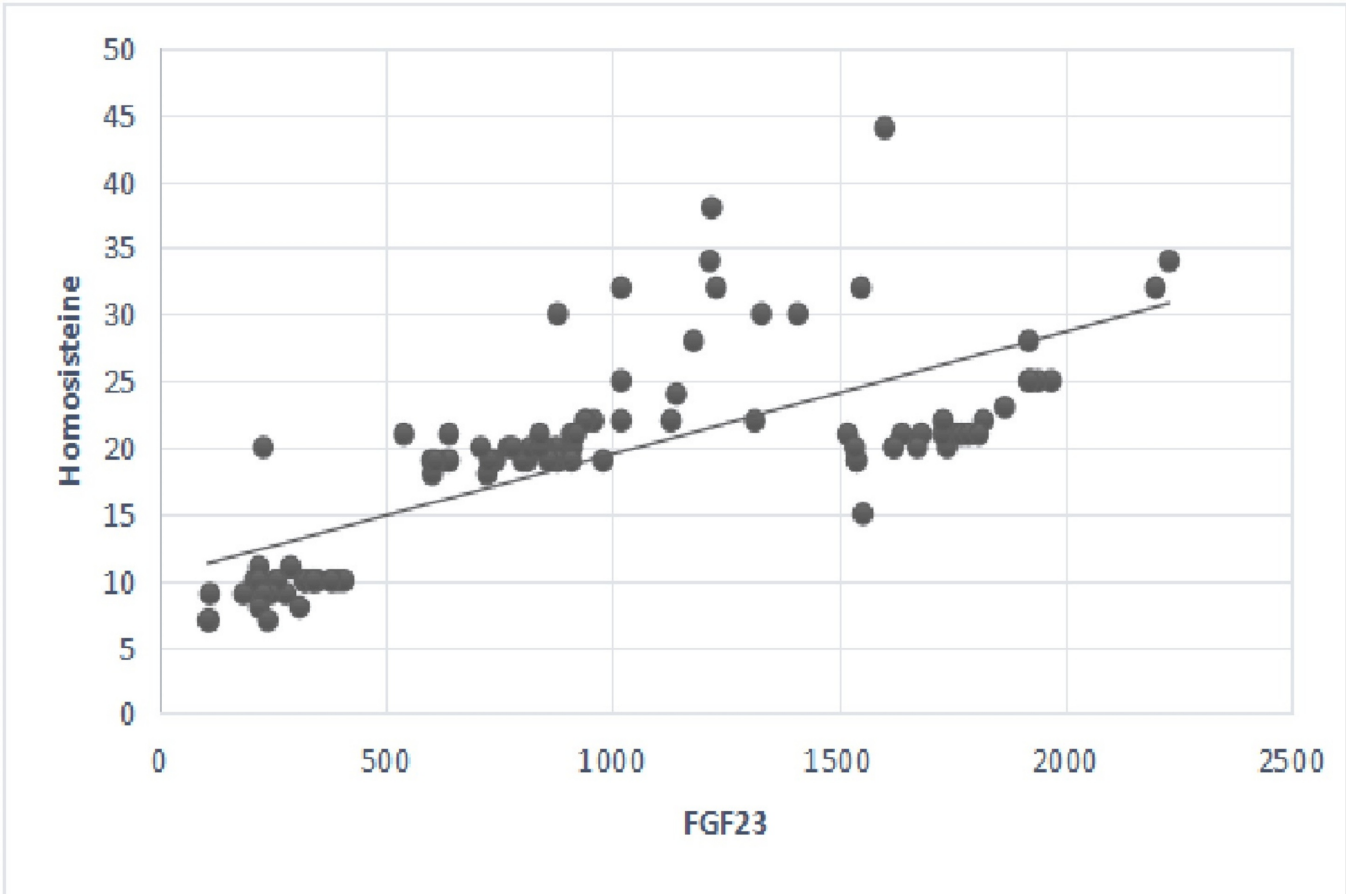
Correlation between serum levels of FGF-23 and serum Hcy rate. p-value <0.05 considered significant; r=0.65, p=0.01. Hcy: homocysteine (umol/ml); FGF-23: fibroblast growth factor 23 (pg/ml).

Additionally, FGF-23 levels were positively linearly associated with serum phosphorus, iPTH, and Hcy levels in the main group. As a result, FGF-23, iPTH, and Hcy showed a linear increasing tendency (Figures 3, 4). 

## Discussion

FGF-23 is a promising biomarker in chronic kidney disease. Its values were presented as one of the important factors for prognosis and morbidity in HD patients with ESRD. Electrolyte imbalances, such as hyperkalemia, hyperphosphatemia, and hypercalcemia, are usually the result of CKD-MBD, which have been associated with elevated higher plasma levels of FGF-23 and PTH [1-5]. FGF-23 is also associated with dialysis-related CVD complications such as cardiorenal syndrome. Levels of FGF-23 show progressive elevation as CKD stages advance [5-9]. New studies are needed to evaluate the validity of repeated FGF-23 value testing in combination with other laboratory predictive biomarker parameters [9-11].

Elevated PTH levels are associated with mortality in HD patients with ESRD. Bone is the target organ for PTH. FGF-23 loses its hormonal effect to suppress PTH secretion due to decreased expression of parathyroid Klotho, and parathyroid gland becomes target organ during absence of a functioning kidney. In response to maintaining normal phosphate balance after kidney function declines (GFR, etc.), PTH and FGF-23 levels increase. PTH and FGF-23 cannot do their phosphaturic effects, resulting in hyperphosphatemia due to further elevations in iPTH and FGF-23 [12-16].

Recent articles have shown that Hcy as a predictive and prognostic marker for CKD HD patients. Hcy was described as one of the non-classical CVD risk factors in CKD patients. Hcy levels have been shown to be elevated in patients withchronic renal failure. Previously positive relation and correlation with some different risk factors were described. Recent articles reported that Hhcy was recognized, with a rate of 85-90%, as an independent risk factor for developing CVD in CKD HD patients [17-20].

Torguet-Escuder et al. evaluated 251 patients with CKD in a cross-sectional study. They described that FGF-23 and iPTH levels progressively increased with the evolution of CKD stages. As a result, FGF-23 was negatively correlated with renal function indicators and positively correlated with PTH and P. Also, Ca values did not change but P rose especially in stage 4 CKD [1]. Zeng et al. investigated 107 cases of CKD patients. FGF-23 showed a positive correlation with blood PTH and Ca levels in CKD HD patients. However, no statistical correlation relation was found with P [2]. Rodelo-Haad et al. reported in the cohort study of CKD HD patients that FGF-23 directly correlated with serum P levels and also correlated inversely with serum Ca [3]. Amin et al. demonstrated that the relationship between Hcy and biochemical parameters such as creatinine, calcium, albumin, and CRP can be used as a predictive and prognostic marker for CKD HD patients in their study [17]. Xie et al. in a prospective cohort study in CKD patients showed that elevated baseline plasma Hcy levels can serve as an independent special biomarker for the prediction of renal function decline and renal failure parameters [18].

There are not many studies and publications in which FGF-23, Hcy, and iPTH values are correlated together with other biochemical laboratory parameters in HD patients with ESRD. In this study, we evaluated the correlation of FGF-23, iPTH, and Hcy levels with other biochemical laboratory parameters in ESRD HD patients from the Azerbaijan population.

Our single-center retrospective study showed the following results. We found that the mean FGF-23 level in the main group was significantly higher than in the control group (p<0.01). This increase may be an important factor for resulting CKD-MBD in HD patients.

It was found that the distribution of male and female in the groups did not differ statistically (p=0.16), as presented in Table 2. It was determined that phosphorus levels differed according to the groups in the study (p=0.01). Phosphorus levels were higher in the study group. Calcium levels did not differ significantly in the main and control groups (p=0.29). iPTH levels differed according to the groups (p=0.01) and were higher in the main group. In the study, it was determined that GFR levels differed according to the groups (p=0.01). GFR levels were lower in the main group (Table 3).

Besides, FGF-23 levels were positively associated with creatinine and negatively with GFR values, as shown in Table 4 and Figures 1, 2 (p=0.01). Also, FGF-23 levels were positively associated with serum phosphorus, iPTH, and Hcy levels, as shown in Table 4 and Figures 3, 4. However, no statistically significant correlation was found with calcium and FGF-23 values (p>0.05). Additionally, FGF-23 values showed a positive linear correlation with iPTH and Hcy in the main patient group compared with the control group (Figures 3, 4).

It has been described as a feedback mechanism between FGF-23, PTH, vitamin D, phosphate, calcium, and Klotho levels. As a result, elevated PTH controls the synthesis of FGF-23 in bones, and decreased renal Klotho expression increases serum P load, which also induces the production of FGF-23. Elevated FGF-23 plasma levels are related to impaired renal functions, as represented by higher levels of creatinine and eGFR <60 mL/min/1.73 m^2^ [1-6,10,15].

The treatment of pathological changes in phosphocalcic metabolism in CKD patients is related to bone health and cardiovascular morbidity and mortality. Elevated FGF-23, iPTH, and Hcy levels, which are found beginning in early phases of CKD as reported before us, may guide to begin earlier treatment strategies to minimize the progression of kidney failure and its effects as risk factors for CVD and MBD. Renal HPT must be treated medically, supplying vitamin D and reducing phosphate intake, and during later stages, calcimimetics must be added. When medical therapy cannot control the hyperparathyroidism, surgical parathyroidectomy considered to be the next treatment [13-16].

Hyperhomocysteinemia was recognized as an effective independent risk factor for CKD. There is a relationship between Hhcy and kidney injury, glomerulosclerosis, tubular atrophy, interstitial fibrosis, and decreased GFR. Stable levels of Hcy in plasma are regulated by the kidneys through compensatory mechanisms of glomerular filtration by upregulating and downregulating different biochemical pathways. Recent studies reported that Hcy is linearly correlated to creatinine, creatinine clearance, and uric acid. Its relationship with biochemical parameters such as calcium, albumin, and CRP can be used as a predictive and prognostic marker for ESRD patients. In contrast, ESRD patients undergoing routine HD aim to lower Hcy clearance, but in reverse inducing more Hhcy. The mechanism of this process is not yet entirely clear [17-20].

FGF-23 concentration reduction must be one of the major targets for therapies in everyday follow-up clinical practice of HD patients. It is primarily advised to achieve a goal of dietary phosphate restriction and using phosphate binders to keep FGF-23 concentrations lower in CKD HD patients. Additionally, non-calcium containing phosphate binders, calcimimetics and HDF are effective ways to decrease FGF-23 levels for patients on HD [22-25]. CVD pathological processes, like cardiorenal syndrome, are the leading cause of death in HD patients with ESRD [26-29].

We have to mention that the lack of information regarding the small patient sample size, urine P excretion, and 1,25 vit D3 levels are the most important limitations of our study. In our study, FGF-23 values ​​were correlated with other biochemical parameters and biomarkers. Evaluating Hcy ​​values ​​individually with similar parameters is one of the limitations of the study. It was suggested that more research should be conducted on this subject. Randomized controlled clinical studies with large patient populations and samples are needed for further research.

There is no single biomarker that can completely predict CKD progression and follow-up. GFR and creatinine are follow-up markers for renal failure prediction. Correlation of FGF-23, iPTH, and Hcy levels together with other laboratory parameters in CKD HD patients may help us for earlier treatment strategies, try to slow patterns of renal failure, and minimize its impacts on the progression to ESRD.

## Conclusions

Values of FGF-23 showed a positive correlation with iPTH, Hcy, P, and creatinine and a negative correlation with GFR. Correlation of FGF-23, iPTH, and Hcy values may be the earliest biomarkers for CKD HD patients. There is no single biomarker that can completely predict and follow-up for CKD progression to ESRD. GFR and creatinine are follow-up markers for renal failure prediction. These parameters together can be used to guide strategies for the prognostic issues and early treatment management in patients with CKD.
